# Gut Microbiota as a Trigger of Accelerated Directional Adaptive Evolution: Acquisition of Herbivory in the Context of Extracellular Vesicles, MicroRNAs and Inter-Kingdom Crosstalk

**DOI:** 10.3389/fmicb.2017.00721

**Published:** 2017-04-20

**Authors:** Marco Romano

**Affiliations:** ^1^Museum für Naturkunde, Leibniz-Institut für Evolutions- und BiodiversitätsforschungBerlin, Germany; ^2^Dipartimento di Scienze della Terra, Sapienza Universita di RomaRome, Italy

**Keywords:** gut microbiota, herbivory, hologenome, inter-kingdom crosstalk, extracellular vesicles, microRNAs

## Abstract

According to a traditional view, the specific diet in vertebrates is one of the key factors structuring the composition of the gut microbiota. In this interpretation, the microbiota assumes a subordinate position, where the larger host shapes, through evolution and its fitness, the taxonomical composition of the hosted microbiota. The present contribution shows how the evolution of herbivory, framed within the new concept of holobiont, the possibility of inter-kingdom crosstalk and its epigenetic effects, could pave the way to a completely reversed interpretation: instead of being passively shaped, the microbiota can mold and shape the general host body structure to increase its fitness. Central elements to consider in this context are the inter-kingdom crosstalk, the possibility of transporting RNAs through nanovesicles in feces from parents to offspring, and the activation of epigenetic processes passed on vertically from generation to generation. The new hypothesis is that the gut microbiota could play a great role in the macroevolutionary dynamics of herbivorous vertebrates, causing directly through host-microbiota dialog of epigenetic nature (i.e., methylation, histone acetylation, etc.), major changes in the organisms phenotype. The vertical exchange of the same microbial communities from parents to offspring, the interaction of these microbes with fairly uniform genotypes, and the socially restricted groups where these processes take place, could all explain the reasons why herbivory has appeared several time (and independently) during the evolution of vertebrates. The new interpretation could also represent a key factor in understanding the convergent evolution of analogous body structures in very distant lineages.

“*Young man says ‘you are what you eat’ - eat well*”

(Dancing With The Moonlit Knight, Selling England by the Pound, Genesis, 1973)

## Introduction

For a long time, microbes associated with eukaryotic organisms were considered in a reductionist way as negative factors able to cause serious disease. Therefore, until relatively recent times, research on microbes was focused on the understanding of disease, in order to cure or better prevent it (Dethlefsen et al., 2007). However, in recent decades this purely antagonistic vision between host and microbes has been totally revolutionized. Just considering *Homo sapiens*, up to 100 trillion microbes colonize the adult (Ley et al., 2008), and the microbial communities associated exhibit a genome with about one hundred times more genes than the human one (Borer et al., 2013). This massive presence of microbiota is not at all passive, but intimately related to biological processes also crucial for host general fitness.

Several studies have clearly shown that the symbiotic relationship with bacterial communities not only increases the overall fitness of the organism, but in several cases are strictly necessary for the survival of the host itself. Gut microbiota are able to extract nutrients useful to the host but otherwise not usable; symbiotic microbes are beneficial to the host’s protection preventing the invasion by pathogens through active stimulation of the immune system, and can promote the differentiation of tissues (Costello et al., 2012). According to some studies, the whole vertebrate adaptive immune system evolved precisely in relation to an increasingly complex symbiosis with microbial communities (Wang et al., 2015). In humans, the alteration of associated microbial communities (i.e., dysbiosis) may cause several clinical disorders, among which are inflammatory diseases, malnutrition, obesity (Costello et al., 2012), diabetes (Wen et al., 2008), and coronary heart diseases (Fava et al., 2006).

According to some pioneering studies (e.g., Troyer, 1984; Lombardo, 2008), the need to horizontally transmit microbial communities ultimately lead to the evolution of sociality among vertebrates; in this context, a progressive development of kin association increases the opportunities of a complete exchange of mutualistic microbes among members of the same restricted group. Subsequent studies have fully demonstrated that the emergence of social activities, in small and circumscribed groups, plays a key role in the structuring and consolidation of shared communities of microbes (e.g., Gilbert, 2015). The bacteria, in addition, have an important role in the generation of specific odor, important for kin recognition and thus for the detection of complex social relationships (Archie and Theis, 2011).

Recently it has been shown that an interaction with microbes may have important influences also in animal development (e.g., Pradeu, 2011), a series of processes typically considered as autonomous, entirely programmed and directed by the sole genome (McFall-Ngai et al., 2013).

In the last decades, thanks to advances in molecular biology and the development of new methodologies (e.g., next-generation sequencing technologies), the taxonomic richness of gut microbiota has been studied in detail, not just in humans but also in several group of vertebrates, from iguanas (e.g., Lankau et al., 2012) to bat families (Phillips et al., 2012) just to mention two cases.

Such constantly increasing studies represent a new critical mass of key information about the intimate microbiota-host relationship along a broad range of animal and plant groups, with possible repercussions in the field of coevolution and macroevolution. Below are discussed the new elements derived by this new mass of data and evidence.

## Host–Microbes Coevolution and the Concepts of Holobiont and Hologenoma

Coevolution between mammals and their indigenous microbial communities has been extensively explored by Ley et al. (2008), based on fecal microbiota of 60 mammalian species including humans. The bacterial diversity is influenced by the diet and phylogenetic position of the host, identifying an increase in diversity (number of taxa) from carnivory to omnivory to herbivory (Ley et al., 2008). Therefore, the non-random association between taxonomic composition in the community of microbes and the type of diet seems very strong evidence in support of a coevolution between host and gut microbiota.

Muegge et al. (2011) show how diet can be a strong driving factor at a large taxonomic scale, demonstrating a clear convergence in the composition of gut microbiota in mammals with the same specific feeding habit. This convergence is confirmed empirically by Delsuc et al. (2014) in myrmecophagous mammals that diverged some 100 million years ago.

Coevolution for millions of years between host and internal microbiota may be considered to represent a new paradigm in molecular biology (e.g., Yeoman et al., 2011; Keenan and Elsey, 2015), proposed and supported specifically for humans (e.g., Muegge et al., 2011), alligators (Keenan and Elsey, 2015), mammals in general (Muegge et al., 2011; McFall-Ngai et al., 2013) and birds (McFall-Ngai et al., 2013). In mammals and birds, the enteric bacteria are characterized by growth optima at ∼40°C, very likely an example of co-evolution with the development of endothermy typical of these animal groups.

Beneficial microbes over the course of time evolved the ability to manipulate different processes in the host, going ultimately to increase their own fitness by increasing the host fitness (Ayres, 2016), an almost didactic example of mutualism, developed in the framework of co-evolution over millions of years. In this context, the importance for the diet of the host-associated microbes was highlighted and stressed up to consider the mutualistic microbiota as an integral part of the phenotype (McFall-Ngai et al., 2013).

Several terms have been proposed in the literature to indicate such ‘indissoluble’ connection between microbial communities and their host: “superorganisms” (e.g., Muegge et al., 2011), “metaorganism” (e.g., Bosch and McFall-Ngai, 2011), “organ system” (e.g., Brown and Hazen, 2015), “metagenome” (e.g., Ley et al., 2008; however, terms considered not totally correct by Bordenstein and Theis, 2015).

This increasing awareness of the close microbe-host connection resulted in the fundamental concepts of ‘holobionts’ and ‘hologenome.’ Bordenstein and Theis (2015) consider animals and plants no longer as perfectly autonomous entities: organisms must be considered as a complex biomolecular network, composed of microbes and the host. This new entity is called the ‘holobiont’ (originally introduced by the ‘visionary’ Lynn Margulis), whereas the whole of host genotypes and microbial communities is defined as ‘hologenome’ (plants and animals are thus considered polygenomic entities). Current biological theories and evolutionary models not taking into account the concepts of holobiont and hologenome, must necessarily be considered as incomplete (Bordenstein and Theis, 2015).

On the evolutionary level, the next great interpretive step was to consider the holobiont as a ‘unit of selection’ in evolutionary processes, starting from the pionieristic intuition by Rosenberg et al. (2009) within the framework of the ‘hologenome theory of evolution.’ The microbial symbionts have a direct effect on holobiont fitness, thus affecting the adaptation, and, therefore, the evolution of higher organisms. According to Rosenberg et al. (2009), in the conditions of rapid shift in environmental parameters, equally rapid changes in the diverse microbial symbiont can lead to a differential survival of the holobiont (with rapid change times unthinkable only on the basis of classic host genome). The fact that the genetic variation occurring in the genomes of both microbial symbiont and host can be vertically passed to the offspring (Bosch and McFall-Ngai, 2011), gives an idea of the importance of this new concept of holobiont as unit of selection in evolutionary studies.

To this already complex framework, a number of key processes must be added, with major repercussions in the epigenetic field: the lateral exchange of genes within the microbiota and between the microbiota and host (i.e., inter-kingdom crosstalk).

## Horizontal Gene Transfer and Inter-Kingdom Crosstalk

Lateral exchanges of genes must be considered a central process in the evolution of microbial genome (Ley et al., 2006). The Horizontal Gene Transfer (HGT) is in fact interpreted by Yeoman et al. (2011) as a real “*hallmark of microbial communities*,” able to drive the microbial evolution (and ecology). An inter-kingdom crosstalk has been shown between humans and bacteria in several studies focusing on the importance of microRNAs. Weiberg et al. (2015) discussed different examples about the important role of sRNAs and RNAi in the communication between eukaryotic and host; the authors show how mobile silencing signals (e.g., sRNAs) can be transferred between host and microorganism in both directions. The circulating exosomal miRNAs can be internalized in recipient cells and then work directly as gene expression regulators (Weiberg et al., 2015). Thus, beyond the classic vertical transfer of genetic information, the HGT by small RNAs (approximately 19–25 nucleotides in length) seems to be a very common process in nature. Such mobile signals can move across kingdoms carrying and spreading silencing information in respect of specific target genes (Han and Luan, 2015). HGT can lead to genetic novelties in the target organisms, and cause phenotypic variation able to allow an access to new trophic resources, and to have improved fitness in shifting environments (Yue et al., 2012).

Crisp et al. (2015) reached the conclusion that HGT “*occurred, and continues to occur, on a previously unsuspected scale in metazoans and is likely to have contributed to biochemical diversification during animal evolution*.” The number of works in the literature on HGT between kingdoms is constantly growing, with exchanges from plants to viruses or animals, bacteria and mammals and many others (e.g., Crisp et al., 2015; Han and Luan, 2015).

Recently Celluzzi and Masotti (2016) presented the interesting perspective that in humans the “other genome” can control our epi-genome, by means of RNAs and other molecules contained in extracellular nanovesicles (produced both by prokaryotes and eukaryotes). Starting from the recent contribution by Celluzzi and Masotti (2016) and Liu et al. (2016) illustrate how the bacteria may interact and alter human epigenome via sRNAs contained within outer membrane vesicles. Intestinal epithelial cells produce microRNAs able to modulate gene expression post-transcriptionally, and to influence the gut microbiota (Liu et al., 2016). The authors demonstrated how extracellular vesicles (EVs) containing microRNAs are present and maintained in fecal samples (Liu et al., 2016). It follows that these vesicles containing ‘information’ (and able to trigger epigenetic changes) can be passed from parents to offspring in species where coprophagy is necessary for the establishment of the gut microbial communities; this process characterizes a large portion of herbivorous animals.

From a macroevolutionary point of view, the central factor to investigate is how the more frequently recognized inter-kingdom communication can have a direct influence on phenotypes subjected to natural selection. The case study of herbivory can be taken as an example of the potential impact of small RNAs transported through nanovesicles in macroevolutionary field.

## Herbivory and Fundamental Importance of Microbial Gut Community

Herbivory represents a special case study in understanding the microbiota communities and the interactions with their host. In fact, in the majority of herbivorous organisms, bacterial populations in the gut are strictly necessary to allow the digestion of celluloses and hemicelluloses (e.g., Hong et al., 2011): i.e., to hydrolyze and ferment plant polymers, which would otherwise be totally indigestible to the host (Lombardo, 2008). *Vice versa*, the host represents the optimal conditions of pH, temperature and moisture for the growth of the microbial population, and provides end-products removal and substrate (Mackie et al., 2004).

The fact that this mutualistic association worked perfectly during vertebrate evolutionary history, possibly leading to a coevolution between microbiota and host, can be inferred indirectly by the numerous independent appearances (on a phylogenetic level) of the herbivorous diet in all vertebrate groups, from reptiles, to birds and mammals. The evolutionary success of this diet is represented simply by the fact that the 80% of extant mammals are herbivores (Ley et al., 2008).

Besides analyzing the current distribution of herbivorous taxa, probably more crucial for a macroevolutionary perspective is the ‘vertical’ study (i.e., phylogenetic) of the appearance of herbivory in vertebrates during the course of geological time.

**FIGURE 1:**
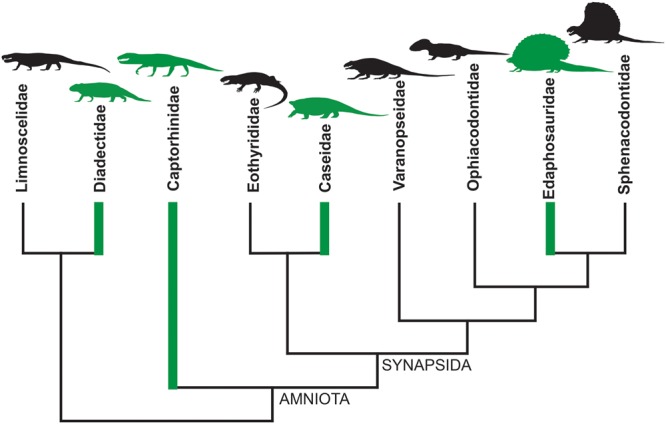
**Simplified cladogram of basal amniotes and their closest relatives.** The green branches indicate that herbivory evolved at least four times independently during the Late Paleozoic, having been recognized within Diadectidae, Captorhinidae, Caseidae, and Edaphosauridae (simplified from Reisz and Sues, 2000, p. 36, Figure 2.12).

An instructive example is represented by the evolutionary history of diets in basal tetrapods at the non-amniote-amniote transition: a period between Late Carboniferous and Early Permian when complex continental ecosystems appeared for the first time on the emerged lands (Pearson et al., 2013; Brocklehurst et al., 2015). Ecosystems reached a ‘modern type’ balanced structure only during the Late Permian, with a high number of herbivorous organisms supporting a smaller number of carnivores (e.g., Hotton et al., 1997). From the Late Carboniferous to Early Permian an herbivorous diet has been inferred for Diadectidae among Diadectomorpha, for Captorhinidae within Permian reptiles, and for Edaphosauridae and Caseidae among Permo-Carboniferous synapsids (Olson, 1968; Reisz, 1986, 2005; Modesto, 1995; Hotton et al., 1997; Reisz and Sues, 2000; Berman et al., 2004; Romano and Nicosia, 2014, 2015; Brocklehurst, 2016; Brocklehurst and Brink, 2017; Romano, 2017). Thus, as early as the Late Paleozoic, obligatory herbivory appeared independently at least four times (**Figure 1**). In addition, just limiting the study to the Permian period, a completely herbivore diet has independently evolved in the Bolosauridae (Reptilia), Pareiasauria (Parareptilia), and among Dinocephalia and Anomodontia within the Therapsida (Reisz and Sues, 2000).

Is there a possible relationship between the gut microbiota and symbiotic convergent evolution of herbivory as early as the first formation of structured ecosystems in the Late Paleozoic? How far can the macroevolutionary influence of symbiotic microbes be extended?

## The Microbiota as a Possible Accelerated Trigger of Epigenetic and Directional Acquisition of Phenotypic Traits

A traditional and widely accepted view is that the specific diet developed in vertebrates is one of the key factors structuring the composition of the gut microbiota (e.g., Ley et al., 2008). Thus, the microbiota assumes a subordinate position: an asymmetrical relationship where the larger host shapes, through evolution and its fitness, the taxonomical content of the hosted microbiota. However, the concept of holobiont, the special case of herbivory, the possibility of inter-kingdom crosstalk and its epigenetic effects, could pave the way to a completely reversed interpretation. In a deliberately provocative way, the question could be as follows: instead of being passively shaped, can the microbiota mold and shape the general host body structure to increase its fitness, possibly in the framework of the selfish gene concept?

In the mutual relationship between the microbiota and the host, natural selection can work to increase the fitness in both directions. However, what is the additional aspect that may drop from the typical evolutionary scheme identified in the new evolutionary synthesis? Surely the inter-kingdom crosstalk and the possibility of transporting RNAs through nanovesicles in feces from parents to offspring, and the activation of epigenetic processes passed on vertically from generation to generation. The processes and elements to be considered are as follows: (1) in herbivorous taxa, gut symbiotic bacteria are essential for breakdown of cellulose and acquisition of nutrients. (2) The need to acquire a proper gut microbiota community has probably led to the evolution of social behavior, which increases the potential for bacterial exchange between parents and offspring and among members of the same narrow social group. (3) In several cases, the communities of bacteria are acquired through ingestion of feces from parents or individuals of the same group. (4) The RNAs contained in nanovesicles are recovered in feces and, through coprophagy, could be passed on to subsequent generations. Once established in the host, microbiota can interact with and alter the host epigenome. The host-microbiota’s dialog, through the mediation of membrane vesicles, can then be passed from parents to offspring, with vertical transmission to subsequent generations. (5) Epigenetic changes might affect the phenotype of the host themselves, with differential development of several organs; for example, the development of very long and massive gut could be stimulated by the host-microbiota dialog, to increase the fitness of the microbes itself (and so consequently that of the host). (6) The genetic variation in the microbiome are orders of magnitude faster than those accumulated in the host genome; evolution finally acts on genetic variation then the changes of microbial origin in the hologenome may be preferred targets for evolution (Bordenstein and Theis, 2015). (7) As mentioned above, the communities of bacteria may have favored the evolution of social behavior. This leads to the structuring of small groups, with a total group (i.e., a ‘core’) genome necessarily more uniform; this should favor the host-microbiota dialog, since the microbiota, passed from parent to offspring, would find a very similar if not superimposable genome to interact with. (8) In small groups of conspecific vertebrates the ‘founder effect’ may be important; the genome is less diluted, and then generation after generation a greater chance of occurrence and fixing of particulates phenotypic traits is expected. Over time, these processes are expected to lead to even greater changes in the general structure of vertebrates. (9) The hologenome theory involves both Darwinism and Lamarckism aspects; the holobiont, in fact, with respect to the concept of evolution in an organism through selection of random mutations, can evolve through adaptive processes (Rosenberg et al., 2009). With respect to individual organisms, thus, the holobiont can evolve through an inheritance of acquired characteristics (Rosenberg et al., 2009).

Within the framework of these elements and processes, the ‘bold’ hypothesis is that the gut microbiota may have (at least in the early stages of transition to a new trophic niche) greater influence in the macroevolutionary dynamics of herbivorous vertebrates, causing directly through host-microbiota dialog of epigenetic nature (i.e., methylation, histone acetylation, etc.), even major changes in the phenotype of the organisms; the driving effect of the process would surely be a direct positive feedback on the fitness of the microbes themselves. The vertical exchange of the same microbial communities from parents to offspring (which guarantees the transgenerational stability in the composition of microbes as number and taxonomic diversity), the interaction of these microbes with fairly uniform or at least compatible genotypes, the socially restricted groups where these processes take place (with greater possibilities of occurrence and affirmation of new phenotypic characters), could all perhaps explain how herbivory has appeared so many times during the evolution of vertebrates; and could also represent a key factor in understanding convergent evolution, within a relatively short time in a geological context, of analogous body structures in very distant lineages.

The possibility of a direct stimulation by bacteria of organ formation and development (such as the gut) might seem excessive and not supported by experimental data. However, Bates et al. (2006) have shown how the zebrafish gut has an unbalanced development in the absence of symbiotic microbiota, due to a stop in differentiation (causing obviously major problems in the absorption of nutrients). The development of the organ restarts if the classic symbiotic microbial community is reintroduced in the sterile organism. This clearly indicates that the gut microbiota in zebrafish plays a key role in cell homeostasis, epithelial maturation and cell-type specification during the crucial development phase (Bates et al., 2006). To this must be added the evidence from recent studies that gut bacterial content can consistently affect the host behavior. Several studies in mice and humans in fact showed how commensal bacteria contribute to brain development and function and can directly affect several complex behaviors (e.g., Collins et al., 2012; Cryan and Dinan, 2012; Hsiao et al., 2013; Tillisch et al., 2013). The emergence of herbivory from a fully carnivorous or omnivorous ancestor must also cover an initial and progressive ‘dislike’ of meat by animals that were otherwise able to obtain energy from this kind of food. Thus, considering the studies cited above, a direct connection and feedback cannot be ruled out between the specific microbiota assortment, the food chosen and taken up (a factor that can be considered in the context of the behavior), the stabilization of a “pro-herbivory” microbiota and its shared metabolome with the host, and again the host behavior influenced by the “herbivore” microbiota.

The hypothesis proposed here, exciting in some ways, but too little explored and very speculative, should be analyzed in-depth, especially on wild animal communities. Many key studies about epigenetics, microbiota composition and inter-kingdom crosstalk have always focused on humans. However, the human species could represent the least suitable case study for this type of purpose, considering that cultural, social and pharmacological aspects can greatly affect the composition of the associated microbiota. Just think of the massive use of artificial antibiotic treatments that undermine or disrupt the microbial composition, a clear ‘technological’ autapomorphy of *Homo sapiens*; to this we must add the chance to travel all over the world and the close contact situations between numerous individuals, such as travel in public transport, big concerts and other large social agglomerations; all elements that should lead to a massive homologation in the content of the microbiota with respect to small family groups of animals often isolated in nature.

The perspective presented in this short contribution would once again lead to the forefront a revival of the much ‘demonized’ Lamarckism and the importance of epigenetics in the evolutionary field. The debate on how to integrate the new discoveries in the field of epigenetics with the classic modern synthesis is still ongoing. According to some authors, the new findings may be totally framed within current evolutionary synthesis (Laland et al., 2014); according to others the new data and evidence strictly require a new set of paradigms and assumptions (e.g., McFall-Ngai et al., 2013; Bordenstein and Theis, 2015). In particular Bordenstein and Theis (2015) speak evocatively of a classic “*eukaryocentricism and nucleocentrism*,” which overlooks the possible central role of microbiology in the evolutionary field, whereas Goldenfeld and Woese (2007) use the expression “molecular reductionism,” for the classical approach dominating the biological field of the twentieth-century.

In light of new discoveries and advancements, probably a new synthesis of evolution based on holobiont and hologenome is strictly necessary and advisable (a “*postmodern synthesis*” in the words of Bordenstein and Theis, 2015). Essentially, this would be a theory or set of theories able to integrate the classical accepted principles by Darwin, Mendel, the modern synthesis, epigenesis and inter-kingdom crosstalk, including also a substantial rethinking of the Biological Species Concept.

## Author Contributions

The author confirms being the sole contributor of this work and approved it for publication.

## Conflict of Interest Statement

The author declares that the research was conducted in the absence of any commercial or financial relationships that could be construed as a potential conflict of interest.

## References

[B1] Archie E. A. Theis K. R. (2011). Animal behaviour meets microbial ecology. *Anim. Behav.* 82 425–436. 10.1016/j.anbehav.2011.05.029

[B2] Ayres J. S. (2016). Cooperative microbial tolerance behaviors in host-microbiota mutualism. *Cell* 165 1323–1331. 10.1016/j.cell.2016.05.049 27259146 PMC4903080

[B3] Bates J. M. Mittge E. Kuhlman J. Baden K. N. Cheesman S. E. Guillemin K. (2006). Distinct signals from the microbiota promote different aspects of zebrafish gut differentiation. *Dev. Biol.* 297 374–386. 10.1016/j.ydbio.2006.05.006 16781702

[B4] Berman D. S. Henrici A. C. Kissel R. A. Sumida S. S. Martens T. (2004). A new diadectid (Diadectomorpha), *Orobates pabsti* from the early permian of central Germany. *Bull. Carnegie Mus. Nat. Hist.* 35 1–36. 10.1098/rsbl.2015.0100

[B5] Bordenstein S. R. Theis K. R. (2015). Host biology in light of the microbiome: ten principles of holobionts and hologenomes. *PLoS Biol.* 13:e1002226 10.1371/journal.pbio.1002226 PMC4540581 26284777

[B6] Borer E. T. Kinkel L. L. May G. Seabloom E. W. (2013). The world within: quantifying the determinants and outcomes of a host’s microbiome. *Basic Appl. Ecol.* 14 533–539. 10.1016/j.baae.2013.08.009

[B7] Bosch T. C. McFall-Ngai M. J. (2011). Metaorganisms as the new frontier. *Zoology* 114 185–190. 10.1016/j.zool.2011.04.001 21737250 PMC3992624

[B8] Brocklehurst N. (2016). Rates and modes of body size evolution in early carnivores and herbivores: a case study from Captorhinidae. *PeerJ* 4:e1555 10.7717/peerj.1555 PMC4715457 26793424

[B9] Brocklehurst N. Brink K. S. (2017). Selection towards larger body size in both herbivorous and carnivorous synapsids during the Carboniferous. *Facets* 2 68–84. 10.1139/facets-2016-0046

[B10] Brocklehurst N. Ruta M. Müller J. Fröbisch J. (2015). Elevated extinction rates as a trigger for diversification rate shifts: early amniotes as a case study. *Sci. Rep.* 5:17104 10.1038/srep17104 PMC4655484 26592209

[B11] Brown J. M. Hazen S. L. (2015). The gut microbial endocrine organ: bacterially derived signals driving cardiometabolic diseases. *Annu. Rev. Med.* 66 343–359. 10.1146/annurev-med-060513-093205 25587655 PMC4456003

[B12] Celluzzi A. Masotti A. (2016). How our other genome controls our epi-genome. *Trends Microbiol.* 24 777–787. 10.1016/j.tim.2016.05.005 27289569

[B13] Collins S. M. Surette M. Bercik P. (2012). The interplay between the intestinal microbiota and the brain. *Nat. Rev. Microbiol.* 10 735–742. 10.1038/nrmicro2876 23000955

[B14] Costello E. K. Stagaman K. Dethlefsen L. Bohannan B. J. Relman D. A. (2012). The application of ecological theory toward an understanding of the human microbiome. *Science* 336 1255–1262. 10.1126/science.1224203 22674335 PMC4208626

[B15] Crisp A. Boschetti C. Perry M. Tunnacliffe A. Micklem G. (2015). Expression of multiple horizontally acquired genes is a hallmark of both vertebrate and invertebrate genomes. *Genome Biol.* 16:50 10.1186/s13059-015-0607-3 PMC4358723 25785303

[B16] Cryan J. F. Dinan T. G. (2012). Mind-altering microorganisms: the impact of the gut microbiota on brain and behaviour. *Nat. Rev. Neurosci.* 13 701–712. 10.1038/nrn3346 22968153

[B17] Delsuc F. Metcalf J. L. Wegener Parfrey L. Song S. J. González A. Knight R. (2014). Convergence of gut microbiomes in myrmecophagous mammals. *Mol. Ecol.* 23 1301–1317. 10.1111/mec.12501 24118574

[B18] Dethlefsen L. McFall-Ngai M. Relman D. A. (2007). An ecological and evolutionary perspective on human–microbe mutualism and disease. *Nature* 449 811–818. 10.1038/nature06245 17943117 PMC9464033

[B19] Fava F. Lovegrove J. A. Gitau R. Jackson K. G. Tuohy K. M. (2006). The gut microbiota and lipid metabolism: implications for human health and coronary heart disease. *Curr. Med. Chem* 13 3005–3021. 10.2174/092986706778521814 17073643

[B20] Gilbert J. A. (2015). Social behavior and the microbiome. *eLife* 4:e07322 10.7554/eLife.07322 PMC4379491 25826451

[B21] Goldenfeld N. Woese C. (2007). Biology’s next revolution. *Nature* 445 369–369. 10.1038/445369a 17251963

[B22] Han L. Luan Y. S. (2015). Horizontal transfer of small RNAs to and from plants. *Front. Plant Sci.* 6:1113 10.3389/fpls.2015.01113 PMC4674566 26697056

[B23] Hong P. Y. Wheeler E. Cann I. K. Mackie R. I. (2011). Phylogenetic analysis of the fecal microbial community in herbivorous land and marine iguanas of the Galápagos Islands using 16S rRNA-based pyrosequencing. *ISME J.* 5 1461–1470. 10.1038/ismej.2011.33 21451584 PMC3160690

[B24] Hotton N. III Olson E. C. Beerbower R. (1997). “Amniote origins and the discovery of herbivory,” in *Amniote Origins* eds Sumida S. S. Martin K. L. M. (San Diego, CA: Academic Press) 207–264.

[B25] Hsiao E. Y. McBride S. W. Hsien S. Sharon G. Hyde E. R. McCue T. (2013). Microbiota modulate behavioral and physiological abnormalities associated with neurodevelopmental disorders. *Cell* 155 1451–1463. 10.1016/j.cell.2013.11.024 24315484 PMC3897394

[B26] Keenan S. W. Elsey R. M. (2015). The good, the bad, and the unknown: microbial symbioses of the American alligator. *Integr. Comp. Biol.* 55 972–985. 10.1093/icb/icv006 25888944

[B27] Laland K. Uller T. Feldman M. Sterelny K. Müller G. B. Moczek A. (2014). Does evolutionary theory need a rethink? *Nature* 514 161–164. 10.1038/514161a 25297418

[B28] Lankau E. W. Hong P. Y. Mackie R. I. (2012). Ecological drift and local exposures drive enteric bacterial community differences within species of Galapagos Iguanas. *Mol. Ecol.* 21 1779–1788. 10.1111/j.1365-294X.2012.05502.x 22369350

[B29] Ley R. E. Hamady M. Lozupone C. Turnbaugh P. J. Ramey R. R. Bircher J. S. (2008). Evolution of mammals and their gut microbes. *Science* 320 1647–1651. 10.1126/science.1155725 18497261 PMC2649005

[B30] Ley R. E. Peterson D. A. Gordon J. I. (2006). Ecological and evolutionary forces shaping microbial diversity in the human intestine. *Cell* 124 837–848. 10.1016/j.cell.2006.02.017 16497592

[B31] Liu S. da Cunha A. P. Rezende R. M. Cialic R. Wei Z. Bry L. (2016). The host shapes the gut microbiota via fecal microRNA. *Cell Host Microbe* 19 32–43. 10.1016/j.chom.2015.12.005 26764595 PMC4847146

[B32] Lombardo M. P. (2008). Access to mutualistic endosymbiotic microbes: an underappreciated benefit of group living. *Behav. Ecol. Sociobiol.* 62 479–497. 10.1007/s00265-007-0428-9

[B33] Mackie R. I. Rycyk M. Ruemmler R. L. Aminov R. I. Wikelski M. (2004). Biochemical and microbiological evidence for fermentative digestion in free-living land iguanas (*Conolophus pallidus*) and marine iguanas (*Amblyrhynchus cristatus*) on the Galápagos Archipelago. *Physiol. Biochem. Zool.* 77 127–138. 10.1086/383498 15057723

[B34] McFall-Ngai M. Hadfield M. G. Bosch T. C. Carey H. V. Domazet-Lošo T. Douglas A. E. (2013). Animals in a bacterial world, a new imperative for the life sciences. *Proc. Natl. Acad. Sci. U.S.A.* 110 3229–3236. 10.1073/pnas.1218525110 23391737 PMC3587249

[B35] Modesto S. (1995). The skull of the herbivorous synapsid *Edaphosaurus boanerges* from the Lower Permian of Texas. *Palaeontology* 30 213–239.

[B36] Muegge B. D. Kuczynski J. Knights D. Clemente J. C. González A. Fontana L. (2011). Diet drives convergence in gut microbiome functions across mammalian phylogeny and within humans. *Science* 332 970–974. 10.1126/science.1198719 21596990 PMC3303602

[B37] Olson E. C. (1968). The family Caseidae. *Fieldiana* 17 225–349.

[B38] Pearson M. R. Benson R. B. J. Upchurch P. Fröbisch J. Kammerer C. F. (2013). Reconstructing the diversity of early terrestrial herbivorous tetrapods. *Palaeogeogr. Palaeoclimatol. Palaeoecol.* 372 42–49. 10.1016/j.palaeo.2012.11.008

[B39] Phillips C. D. Phelan G. Dowd S. E. McDonough M. M. Ferguson A. W. Delton Hanson J. (2012). Microbiome analysis among bats describes influences of host phylogeny, life history, physiology and geography. *Mol. Ecol.* 21 2617–2627. 10.1111/j.1365-294X.2012.05568.x 22519571

[B40] Pradeu T. (2011). A mixed self: the role of symbiosis in development. *Biol. Theory* 6 80–88. 10.1007/s13752-011-0011-5

[B41] Reisz R. R. (1986). “Pelycosauria,” in *Handbuch der Paläoherpetologie* Vol. 17A ed. Wellnhofer P. (Stuttgart: Gustav Fischer Verlag) 102.

[B42] Reisz R. R. (2005). *Oromycter*, a new caseid from the Lower Permian of Oklahoma. *J. Vertebr. Paleontol.* 25 905–910. 10.1671/0272-4634(2005)025[0905:OANCFT]2.0.CO;2

[B43] Reisz R. R. Sues H. D. (2000). “Herbivory in late Paleozoic and Triassic terrestrial vertebrates,” in *Evolution of Herbivory in Terrestrial Vertebrates* ed. Sues H.-D. (Cambridge: Cambridge University Press) 9–41. 10.1017/CBO9780511549717.003

[B44] Romano M. (2017). Long bone scaling of caseid synapsids: a combined morphometric and cladistic approach. *Lethaia.* 10.1111/let.12207

[B45] Romano M. Nicosia U. (2014). *Alierasaurus ronchii*, gen. et sp. nov., a caseid from the Permian of Sardinia, Italy. *J. Vertebr. Paleontol.* 34 900–913. 10.1080/02724634.2014.837056

[B46] Romano M. Nicosia U. (2015). Cladistic analysis of Caseidae (Caseasauria, Synapsida): using Gap-Weighting Method to include taxa based on poorly known specimens. *Palaeontology* 58 1109–1130. 10.1111/pala.12197

[B47] Rosenberg E. Sharon G. Zilber-Rosenberg I. (2009). The hologenome theory of evolution contains Lamarckian aspects within a Darwinian framework. *Environ. Microbiol.* 11 2959–2962. 10.1111/j.1462-2920.2009.01995.x 19573132

[B48] Tillisch K. Labus J. Kilpatrick L. Jiang Z. Stains J. Ebrat B. (2013). Consumption of fermented milk product with probiotic modulates brain activity. *Gastroenterology* 144 1394–1401. 10.1053/j.gastro.2013.02.043 23474283 PMC3839572

[B49] Troyer K. (1984). Microbes, herbivory and the evolution of social behavior. *J. Theor. Biol.* 106 157–169. 10.1016/0022-5193(84)90016-X

[B50] Wang J. Kalyan S. Steck N. Turner L. M. Harr B. Künzel S. (2015). Analysis of intestinal microbiota in hybrid house mice reveals evolutionary divergence in a vertebrate hologenome. *Nat. Commun.* 6:6440 10.1038/ncomms7440 PMC4366507 25737238

[B51] Weiberg A. Bellinger M. Jin H. (2015). Conversations between kingdoms: small RNAs. *Curr. Opin. Biotechnol.* 32 207–215. 10.1016/j.copbio.2014.12.025 25622136 PMC4387066

[B52] Wen L. Ley R. E. Volchkov P. Y. Stranges P. B. Avanesyan L. Stonebraker A. C. (2008). Innate immunity and intestinal microbiota in the development of Type 1 diabetes. *Nature* 455 1109–1113. 10.1038/nature07336 18806780 PMC2574766

[B53] Yeoman C. J. Chia N. Yildirim S. Miller M. E. B. Kent A. Stumpf R. (2011). Towards an evolutionary model of animal-associated microbiomes. *Entropy* 13 570–594. 10.3390/e13030570

[B54] Yue J. Hu X. Sun H. Yang Y. Huang J. (2012). Widespread impact of horizontal gene transfer on plant colonization of land. *Nat. Commun.* 3:1152 10.1038/ncomms2148 PMC3493653 23093189

